# Functional Imaging with Reinforcement, Eyetracking, and Physiological Monitoring

**DOI:** 10.3791/992

**Published:** 2008-11-13

**Authors:** Vincent Ferrera, Jack Grinband, Tobias Teichert, Franco Pestilli, Stephen Dashnaw, Joy Hirsch

**Affiliations:** Department of Neuroscience, Columbia University; Department of Psychiatry, Columbia University; Department of Radiology, Columbia University

## Abstract

We use functional brain imaging (fMRI) to study neural circuits that underlie decision-making.  To understand how outcomes affect decision processes, simple perceptual tasks are combined with appetitive and aversive reinforcement.  However, the use of reinforcers such as juice and airpuffs can create challenges for fMRI.  Reinforcer delivery can cause head movement, which creates artifacts in the fMRI signal.  Reinforcement can also lead to changes in heart rate and respiration that are mediated by autonomic pathways.  Changes in heart rate and respiration can directly affect the fMRI (BOLD) signal in the brain and can be confounded with signal changes that are due to neural activity.  In this presentation, we demonstrate methods for administering reinforcers in a controlled manner, for stabilizing the head, and for measuring pulse and respiration.

**Figure:**
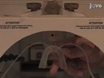


## Protocol

### Equipment Setup

The first step in running and fMRI experiment is setting up and checking the equipment.  These steps can be done in any order.

Signal conditioning - The scanner is located in an electrically and magnetically shielded room.  All electrical signals going from the control room to the scanner room pass through a filter panel to remove any frequencies that could create an artifact in the MR image.

Airpuff – The airpuff device delivers a controlled puff of fixed pressure and duration.  When directed toward the eye, this is aversive but non-traumatic.  The pressure regulator (WPI Pneumatic PicoPump) to a source of compressed air, which could be an air tank but we use house air.  The pressure is adjusted to 30 psi.  The timing is adjusted to deliver a 50 msec puff. The solenoid valve is controlled by a computer signal.  The output of the pressure regulator is delivered to the subject through a 1/6th inch plastic tube (Tygon).  It is important that the tube not be too long or there will be a delay between the solenoid opening and the time that the compressed air arrives at the end of the tube.  For this reason, the pressure regulator sits inside the scanner room and the control signals are fed through a filter panel to ensure that there are no imaging artifacts.

Juice – The juice dispenser controls the size and number of juice drops delivered to the mouth by a plastic tube.  The juice dispenser consists of a reservoir, a computer-controlled solenoid valve, and a long tube that delivers juice to the subject.  All the electronic components are outside the scanner room and therefore introduce no artifacts in the MR signal.  The juice dispenser (Crist Instruments) is rinsed twice by filling reservoir and opening solenoid valve.  Then it is filled the with subject’s preferred beverage.  The system is tested prior to scanning by running a program that causes the computer to generate a stream of test pulses that results in several drops of juice being delivered to the subject.  The subject verifies that the juice is flowing.

Bite bar – Head movement creates several artifacts in the fMRI image.  In this experiment, subjects receive airpuffs which can cause reflexive movements, and juice which requires swallowing.  To minimize head movement, a bitebar is used to stabilize the head.  The bitebar is attached to the RF head coil.  Each subject has a custom-fitted mouthpiece made from thermoplastic material.

Pulse oximeter – FMRI measures blood oxygenation (BOLD).  Blood oxygenation in the brain can be affected by neuronal activity, but it can also be affected by the heartbeat.  To measure changes in oxygenation related to the cardiac cycle, we use a pulse oximeter to measure blood oxygenation in the fingertip using an infrared sensor.  The pulse oximeter is MR compatible and its output signal is fed through a filter panel to the control room where it is digitized and stored on computer.

Respiratory gas monitor – Blood oxygenation can also be affected by respiration.  We measure respiration with a respiratory gas monitor that measures expired CO2 levels. The RGM is MR compatible and its output signal is fed through a filter panel to the control room where it is digitized and stored on computer.

Visual stimulation - A back-projection panel is located at one end of the scanner bore close to the subject head location. Subjects look to the back projection screen through a mirror mounted on top of the head coil. Images are projected onto the back projection screen by an LCD projector located outside the scanner room. The light beam from the projector is directed inside the scanner room through a RF shielded hose.

Eye tracking – It is important to know where the subject is looking for several reasons.  Eye movements affect the position of visual stimuli on the retina, which affects visual responses in the brain.  Eye movements can also be used as a means of having the subject indicate a behavioral response.  We use a method called “infrared video oculography” to track the eyes.  In this method, an infrared camera is used to track the pupil.  The infrared emitter and camera are built into a pair of specially designed goggles.  These are the same goggles that provide visual stimulation.  The data from the goggles is processed by a dedicated computer that converts images of the eye into analog signals for horizontal and vertical eye position.

### Subject Preparation 

Documentation and Consent – Before scanning, each subject must fill out several forms regarding safety and consent.

Experimental Plan Sheet – The plan sheet specifies the experimental protocol and the pulse sequences and the order in which they are run.

Physical Preparation – When the subject is ready to be scanned, one of the first things they do is put in ear plugs to protect their hearing from the loud and high frequency scanner noise.  They also put on MR-compatible headphones to both communicate with the subjects in the scanner and to deliver instructions and feedback during the scanning session.

The subject puts in their mouthpiece, which includes 1/16^th^ inch lines for juice delivery and respiratory gas monitoring.

The subject puts on and adjust the MR compatible goggles used for visual stimulation and eye movement monitoring. 

The subject lies on the scanner bed and the RF coil is positioned over their head. The mouthpiece (bitebar) is attached to the RF coil to prevent head movement.

The airpuff tubing (1/6 inch tygon) is positioned near one eye using flexible tubing (loc-line).

The pulse-oximeter finger clip is attached to one finger and secured with tape.

### Scanning Procedure

Pulse sequences:

*Structural:* We use T1-weighted images (structural images) that give a clear definition of the morphology of the subjects’ brain.  The subject lies passively and remains as still as possible during this phase, which lasts about 10 minutes.

*Functional:* We use T2-weighted EPI sequences (functional images) to show changes in blood oxygenation that are correlated with neural activity.  It is during this phase that the subject is exposed to the juice, airpuff, and auditory stimuli.

*Connectivity:* We used diffusion-weighted images (DWI) to determine structural connectivity between brain regions.  The subject simply lies motionless during this phase.

